# Sonochemical preparation of SnS and SnS_2_ nano- and micropowders and their characterization

**DOI:** 10.1016/j.ultsonch.2021.105594

**Published:** 2021-05-20

**Authors:** Grzegorz Matyszczak, Paweł Jóźwik, Emilia Polesiak, Małgorzata Sobieska, Krzysztof Krawczyk, Cezariusz Jastrzębski, Tomasz Płociński

**Affiliations:** a Department of Chemical Technology, Faculty of Chemistry, Warsaw University of Technology, Noakowskiego Street 3, 00-664 Warsaw, Poland; b Faculty of Advanced Technologies and Chemistry, Military University of Technology, ul. gen. Sylwestra Kaliskiego 2, 00-908 Warsaw, Poland; c Faculty of Physics, Warsaw University of Technology, Koszykowa street 75, 00-662 Warsaw, Poland; d Faculty of Materials Science and Engineering, Warsaw University of Technology, Wołoska Street 141A, 02-507 Warsaw, Poland

**Keywords:** Tin(II) sulfide, Tin(IV) sulfide, Nanopowder, Micropowder, Sonochemistry

## Abstract

Sonochemical production of tin(II) and tin(IV) sulfides is investigated. Different conditions of syntheses are examined: used solvent (ethanol or ethylenediamine), source of tin (SnCl_2_ or SnCl_4_), the molar ratio of thioacetamide to the tin source, and time of sonication. The obtained powders are characterized by the X-ray diffraction method (PXRD), scanning electron microscopy (SEM), scanning transmission electron microscopy (STEM), energy-dispersive X-ray spectroscopy (EDX), and the Tauc method. Raman and FT-IR measurements were performed for the obtained samples, which additionally confirmed the crystallinity and phase composition of the samples. The influence of experimental conditions on composition (is it SnS or SnS_2_), morphology, and on the bandgap of obtained products is elucidated. It was found that longer sonication times favor more crystalline product. Each of bandgaps is direct and most of them show typical values – c.a. 1.3 eV for SnS and 2.4 eV for SnS_2_. However, there are some exceptions. Synthesized powders show a variety of forms such as needles, flower-like, rods, random agglomerates (SnS_2_) and balls (SnS). Using ethanol as a solvent led to powders of SnS_2_ independently of which tin chloride is used. Sonochemistry in ethylenediamine is more diverse: this solvent protects Sn^2+^ cations from oxidation so mostly SnS is obtained, while SnCl_4_ does not produce powder of SnS_2_ but Sn(SO_4_)_2_ instead or, at a higher ratio of thioacetamide to SnCl_4_, green clear mixture.

## Highlights

• Sonochemical synthesis of tin sulfides is investigated.
• Sonochemistry widely varies with synthesis conditions.
• Different experimental conditions lead to very diversified morphology of powder.
• Energy bandgap of tin sulfide powders strongly depends on experimental conditions.

## Graphical abstract

**Figure:**
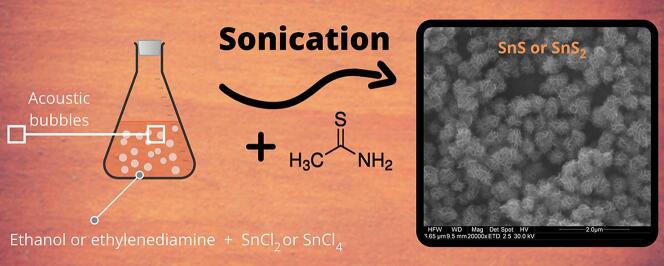

## 1 Introduction

The chemistry of sulfide tin compounds is very rich and interesting. It is well known that tin together with sulfur forms three sulfides: SnS, SnS_2_, and Sn_2_S_3_ [1]. The last one is, in fact, a mixed-valence compound and is a 1:1 mixture of SnS and SnS_2_ [1]. There are some reports on another mixed-valence tin sulfide, Sn_3_S_4_, however, this compound has not been obtained so far in the form of single crystals and its crystal structure is not resolved [2], [3]. Sn_3_S_4_ is typically produced as nanopowders and composites [2], [3]. The family of mixed-valence tin sulfides has been recently extended and studied theoretically using particle swarm optimization package (CALYPSO) combined with first-principles energetic calculations [4]. It was found that the Sn_3_S_4_ shows marginal stability concerning decomposition into SnS and SnS_2_, while other studied compositions - Sn_3_S_5_, Sn_4_S_5_, Sn_4_S_7_, Sn_5_S_6_, Sn_5_S_7_, Sn_5_S_8_, Sn_5_S_9_, Sn_6_S_7_, Sn_7_S_8_ - may be metastable under ambient conditions, with slightly positive formation enthalpies [4].

In the SnS_2_ crystal structure, Sn(IV) coordinates to six S^2-^ ions in regular octahedral [5]. In SnS, Sn(II) coordinates to three S^2-^ ions, with a stereochemically active lone pair of electrons occupying the last tetrahedral site [5]. In tin sulfide-based materials the local geometry around a tin center may vary from trigonal pyramidal, to tetrahedral, trigonal bipyramidal and octahedral, and around sulfur from the terminal, V-shaped to trigonal pyramidal [6]. The oxidation state may take +2 and +4 for tin and −2, −1, 0 for sulfur [6]. The chemistry of tin sulfides is further enriched by the ability of sulfur to the catenation [6]. The catenation length can be as short as in S_2_ and as long as in a polymeric sulfur chain, which may contain more than 200 000 sulfur atoms [7]. Chemistry of tin sulfides is even much more complicated because tin(IV) can form complex anions: monomeric SnS_4_^4-^, and dimeric Sn_2_S_6_^4-^ (edge-sharing) and Sn_2_S_7_^6-^ (corner-sharing) (which are products of condensation of monomeric SnS_4_^4-^ anions) [8], [9], [10]. There are even tetrameric anion Sn_4_S_10_^4-^ which consists of four corner-sharing SnS_4_ tetrahedral units [6].

Tin(II) sulfide is an attractive material for applications in photovoltaics, photonics, and optoelectronics (solar cell devices, sensors, batteries, etc.) [1], [11], [12]. SnS has a bandgap of 1.1–1.5 eV, a high optical absorption coefficient, and p-type conductivity while Sn_2_S_3_ and SnS_2_ display n-type conductivity arising from the dominant sulfur vacancy associated with the Sn(IV) oxidation state [5]. Possible applications of tin sulfides include anodes for sodium and lithium storage (SnS_2_-graphene nanosheets), ultrafast broadband photodetector (SnS), and the detection of ethanol (SnS/SnS_2_ nanoparticles) [13], [14], [15]. Many methods have been used to prepare tin(II) and tin(IV) sulfide nanostructures: solvothermal and hydrothermal routes, hot injection methods, polyol methods, and aqueous solution methods [16], [17], [18], [19], [20], [21], [22], [23].

The hot-injection method needs to use a toxic high-boiling solvent (e. g. oleylamine) which makes this method not suitable for large-scale production. The solvothermal synthesis typically uses high-boiling solvents or toxic materials. A good alternative to the above methods is a sonochemical synthesis which gives promise of effective large-scale production of low-cost nano- and microparticles under mild and environment-friendly conditions [24]. In sonochemistry, molecules undergo chemical reactions caused by powerful ultrasound radiation. The main events in such reactions are the creation, growth, and collapse of a bubble formed in the liquid. Bubbles grow through the diffusion of solute vapor into their volume. Then the collapse of a bubble occurs when it reaches its maximum size. One of many theories says that upon the collapse of a bubble very high temperatures (5000–25000 K) and pressures (order of magnitude 1000 atm) are obtained [25], [26].

Sonochemistry is neat in the synthesis of many simple and complex chalcogenides, e.g. ZnS, Sb_2_S_3_, HgSe, CdS, PbS, and Cu_2_ZnSnS_4_ [24], [27]. Many different conditions of synthesis have been investigated, such as solvent, precursors and chalcogenide source, due to the influence on the properties of the obtained powder [27]. For example, In_2_S_3_ in the form of submicron cage-like structures have been obtained from the ethanol solution of InCl_3_ and it was shown that longer time of sonication decreases the energy band gap and increases the average particle size of powder [28]. Sonochemical synthesis was further improved by combination with electrochemistry [29]. Such an approach creates sonoelectrochemistry, which allows production of nanomaterials with controlled sizes and shapes [30], [31].

Sonochemistry was also applied in the synthesis of SnS and SnS_2_ nanoparticles [32], [33], [34], [35], [36]. Sonochemically synthesized SnS was utilized in the photodegradation of Methyl Blue showing high photocatalytic and photovoltaic activity, while sonochemically obtained nanoparticles of SnS_2_ demonstrated antimicrobial and antioxidant properties [33], [34], [35]. Another example of application of sonochemically synthesized nano- and microparticles of SnS and SnS_2_ is the modification of cathodes for the electro-Fenton process [37].

This study concerns the influence of solvent, sonication time, reagents, and molar ratio of thioacetamide to tin chloride on the morphology, composition, and energy bandgap of obtained powders in the sonochemical syntheses. As a solvent non-chelating ethanol (C_2_H_5_OH) and chelating ethylenediamine (C_2_H_4_(NH_2_)_2_) are used, while as a tin source SnCl_2_ and SnCl_4_ are used. The main novelty of presented study is the investigation of wide range of different experimental conditions of sonochemical syntheses, including many combinations that were not investigated before. There is a lack of such comprehensive studies on this subject.

## 2 Materials and Methods

### 2.1 Material and reagents

All chemicals were pure for analysis (producer: POCH). For sonochemical syntheses, SnCl_2_·2H_2_O, SnCl_4_·5H_2_O, and thioacetamide (TAA) were used as reagents, and ethanol and ethylenediamine were used as solvents. For purification of obtained suspensions ethanol was used. Trichloromethane was used for the preparation of suspensions for UV–Vis spectrophotometry investigation.

### 2.2 Sonochemical syntheses

The sonochemical syntheses were conducted in conical flasks of 50 ml volume in an ultrasonic cleaner (Sonic-33 digital version, Polsonic) generating an ultrasound of 40 kHz frequency with power 2000 W. The acoustic power determined calorimetrically was 38 W/L. 20 ml of solvent was measured with a graduated cylinder and placed in a flask. Weighed reagents were placed in the solvent in the conical flask and the mixture has been stirred magnetically for 30 min. Then flasks were closed with glass stoppers and placed in the ultrasonic cleaner in the depth equal to the level of liquid in the flasks. Ultrasound irradiation was applied for 100–230 min. See Table 1 for information about each of the syntheses.

**Table 1:** Experimental conditions for each synthesis and information about obtained products (results of corresponding investigations). “A” means “amorphous”.

No.	Tin source	Solvent	Sonication time [min]	TAA:tin source ratio	Tin source amount [mg]	TAA amount [mg]	Powder color	XRD	EDX [Sn:S atomic ratio]	Energy bandgap [eV]
1	SnCl_2_	Ethanol	100	2.5	451	376	Orange	A	Sn_3_S_4_, 0.787	2.42
2	SnCl_2_	Ethanol	160	2.5	451	376	Orange	SnS_2_	SnS_2_, 0.554	2.12
3	SnCl_2_	Ethanol	230	1	451	150	Orange	SnS_2_	SnS, 0.947	2.42
4	SnCl_2_	Ethanol	100	3.5	451	526	Orange	SnS_2_	Sn_2_S_3_, 0.651	2.36
5	SnCl_2_	Ethanol	160	3.5	451	526	Orange	SnS_2_	Sn_2_S_3_, 0.664	1.96
6	SnCl_4_	Ethanol	100	2.5	701	376	Orange	SnS_2_	SnS_2_, 0.522	1.79
7	SnCl_4_	Ethanol	160	2.5	701	376	Orange	SnS_2_	SnS_2_, 0.575	1.65
8	SnCl_4_	Ethanol	100	3.5	701	526	Orange	A	SnS_2_, 0.561	2.35
9	SnCl_4_	Ethanol	160	3.5	701	526	Orange	A	–	1.73
10	SnCl_2_	Ethylenediamine	100	2.5	451	376	Dark brown	SnS	SnS, 0.804	1.70
11	SnCl_2_	Ethylenediamine	160	2.5	451	376	White	SnS_2_	SnS_2_, 0.510	2.56
12	SnCl_2_	Ethylenediamine	100	3.5	451	526	Dark brown	SnS	SnS, 0.904	1.29
13	SnCl_2_	Ethylenediamine	160	3.5	451	526	Dark brown	SnS	SnS, 1.019	0.99
14	SnCl_4_	Ethylenediamine	100	2.5	701	376	White	Sn(SO_4_)_2_ + SnCl_2_	–	1.8
15	SnCl_4_	Ethylenediamine	160	2.5	701	376	White	Sn(SO_4_)_2_	–	2.84
16	SnCl_4_	Ethylenediamine	100	3.5	701	526	No powder – green mixture	–	–	–
17	SnCl_4_	Ethylenediamine	160	3.5	701	526	No powder – a green mixture	–	–	–

The purification has been carried out with the following procedure: after reaction, mixture was centrifuged and the supernatant was removed; then the sediment was suspended in fresh ethanol (20 ml) in the ultrasonic cleaner for 15 min; obtained suspension has been centrifuged, supernatant was removed and the sediment was suspended in fresh ethanol (20 ml) in the ultrasonic cleaner for 15 min (this point was repeated 2 times). Finally, a suspension in ethanol is obtained. For the preparation of suspension in trichloromethane for UV–Vis measurements, the described above procedure of purification was applied using CHCl_3_, instead of ethanol, starting from suspension in ethanol.

### 2.3 UV–Vis spectrophotometry

The absorbance spectra of diluted transparent suspensions in CHCl_3_ of synthesized powders were recorded within the wavelength range of 400–1100 nm using a spectrophotometer Model Perkin-Elmer Lambda 20.

### 2.4 SEM and EDX investigations

For the SEM and EDX investigations, a portion of suspension of powder in ethanol was placed in a glass test-tube. The ethanol was then effortlessly evaporated under a laboratory hood. Obtained dry powder was then investigated.

The examination of the surface of obtained powders was performed by scanning electron microscope Quanta 3D FEG using a backscattered type detector.

### 2.5 STEM investigations

The STEM investigations were performed on Cs corrected Hitachi HD2700 microscope. The powder samples were deposited on holey carbon film from suspensions in ethanol, and after drying in room temperature cleaned in low energy plasma cleaner. The observations were taken at 200 kV accelerating voltage, by using SE, BF-STEM and HAADF detectors. Structural analysis was done on fast Fourier transformations of high resolution BF-STEM images.

### 2.6 Powder XRD investigations

Each sample has been prepared by instilling of suspension of powder in ethanol on a microscope slide and effortless evaporation of the solvent under a laboratory hood. Diffractometer RTG HZG-4 was used for analyses.

### 2.7 Raman spectroscopy

The micro-Raman Spectroscopy measurements were carried out by using Renishaw’s inVia Reflex Spectrometer. The Raman spectra were collected at room temperature and normal conditions; in backscattering geometry with the 633 nm line of a He-Ne–ion laser. The studies were performed to investigate microstructural phase homogeneity of the samples.

### 2.8 FTIR spectroscopy

The Fourier-transform infrared spectroscopy measurements were done using NICOLET 6700 FT-IR spectrometer. Samples were dried under laboratory hood and measured in KBr pellets.

## 3 Results and discussion

The data about each conducted synthesis and basic information on products are presented in Table 1.

**Fig. 1:**
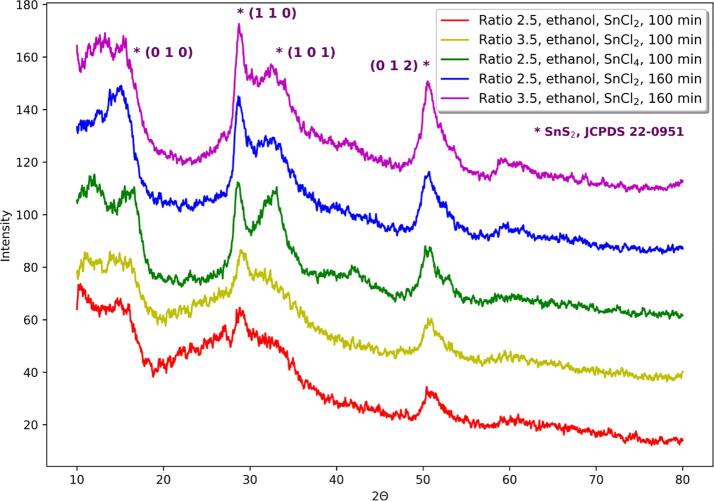
Comparison of diffractograms of obtained powders in sonochemical syntheses with varying sonication time and tin source, using ethanol as a solvent.

Each synthesis in ethanol led to the formation of SnS_2_, which is confirmed by XRD, EDX, and Tauc method investigations. Also, the color of the obtained powders is in agreement with the typical color of SnS_2_. The direct bandgap value of bulk SnS_2_ is about 2.38 eV so our results, laying in range 2.12–2.42 eV, are in good agreement with this value [38]. However, there are two exceptions when the energy bandgap of powders obtained in the syntheses starting from SnCl_4_ with TAA:SnCl_4_ molar ratio 2.5 were found to be 1.79 and 1.65 eV for 100 and 160 min long reactions respectively, what is unusual for SnS_2_. It should be emphasized that XRD and EDX investigations, as well as the color of the powder, confirm that this product is SnS_2_. From results for syntheses using ethanol longer sonication times lead to more crystalline product (Fig. 1). Apart from TAA:SnCl_2_ molar ratio (2.5 or 3.5), 100 min is not enough for the production of powder with even poor crystallinity. Although, changing tin source from SnCl_2_ to SnCl_4_ allows to obtain, in 100-minute synthesis, powder with crystallinity comparable to the crystallinity of product synthesized starting from SnCl_2_ applying longer sonication time (Fig. 1).

**Fig. 2:**
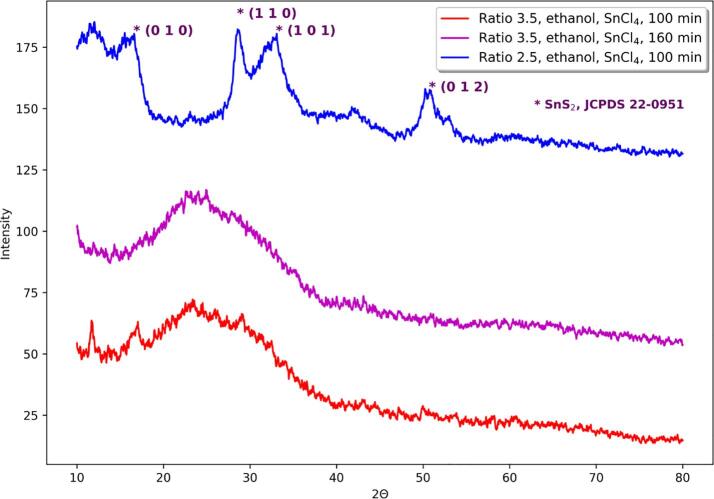
Comparison of diffractograms of obtained powders in sonochemical syntheses with varying sonication time and TAA:tin source molar ratio, using ethanol as a solvent and SnCl_4_ as a tin source.

Moreover, it is interesting that in the case of using SnCl_2_ as a tin source, longer sonication time favors more crystalline products apart from TAA:SnCl_2_ molar ratio while this effect is not observed when starting from SnCl_4_ at TAA:SnCl_4_ ratio 3.5 (Fig. 2).

Sonochemistry in ethylenediamine is more intriguing because not every reaction led to SnS_2_. Syntheses starting from SnCl_4_ at TAA:SnCl_4_ molar ratio 2.5 yielded tin(IV) sulfate Sn(SO_4_)_2_ as revealed by PXRD investigations. In the product of 100 min long synthesis there is still some tin chloride present. Moreover, syntheses starting from SnCl_4_ at TAA:SnCl_4_ ratio 3.5 gave no product. The stable, green and perspicuous mixture is formed instead and even 160 min of sonication is not enough to observe the generation of powder.

**Fig. 3:**
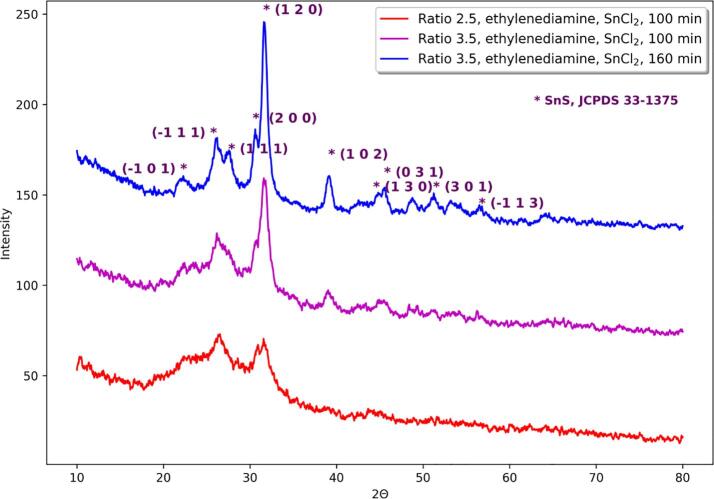
Comparison of diffractograms of obtained powders in sonochemical syntheses with varying sonication time and TAA:tin source molar ratio, using ethylenediamine as a solvent and SnCl_2_ as a tin source.

Syntheses in ethylenediamine starting from SnCl_2_ led mainly to the formation of SnS. However at TAA:SnCl_2_ molar ratio 2.5 after 1 h of additional sonication SnS turns to SnS_2_. This is not observed at TAA:SnCl_2_ molar ratio 3.5. As in the case of using ethanol as a solvent, longer sonication time increases the crystallinity of obtained powder (Fig. 3). Increasing the TAA:SnCl_2_ molar ratio also improves the crystallinity (Fig. 3).

The Hall-Williamson method was applied to analyze the mean particle sizes and strains in sonochemically prepared powders of tin sulfides, according to the following equation:

$$\frac{\beta_{\mathrm{hkl}}cos\theta }{\lambda }=\frac{K}{D}+\varepsilon \left(\frac{4sin\theta }{\lambda }\right)$$

The strain ε is determined as a slope of linear trend line in the plot of $\frac{\beta_{\mathrm{hkl}}cos\theta }{\lambda }$ vs $\frac{4sin\theta }{\lambda }$, and the mean particle size D is calculated as reciprocal of intercept value. β_hkl_ denotes full-width at half maxima of diffraction peak, θ is the diffraction angle and λ is the wavelength of applied X-rays (here it is 1.54 Å). Due to poor quality of powder diffractograms, for SnS_2_ samples only indexes (1 1 0) and (0 1 2) were taken into account, as well as indexes (−1 1 1), (1 2 0), and (1 0 2) for SnS samples. This mean that estimated values of sizes and strains should be considered qualitatively (Table 2). The analysis by Hall-Williamson method reveals that strains in sonochemically prepared SnS_2_ powders are negative, while in the SnS powders they are positive. Moreover, the mean particle sizes of SnS powders are generally greater than SnS_2_ ones. From Table 2 one can also see that there is no clear correlation between sonication time, TAA:tin source ratio, and mean particle sizes in obtained samples.

**Table 2:** Mean particle sizes and strains for different SnS and SnS_2_ sonochemically obtained powders determined using the Hall-Williamson analysis basing on Uniform Deformation Model.

Tin source	TAA:tin source ratio	Solvent	Sonication time [min]	Mean particle size [nm]	Strain
SnCl_2_	2.5	Ethanol	100	2.56	−0.185
SnCl_4_	2.5	Ethanol	100	0.88	−0.478
SnCl_2_	3.5	Ethanol	100	0.83	−0.509
SnCl_2_	2.5	Ethanol	160	0.73	−0.571
SnCl_2_	3.5	Ethanol	160	1.27	−0.357
SnCl_2_	2.5	Ethylenediamine	100	4.83	0.636
SnCl_2_	3.5	Ethylenediamine	100	2.61	0.482
SnCl_2_	3.5	Ethylenediamine	160	3.10	0.454

**Fig. 4:**
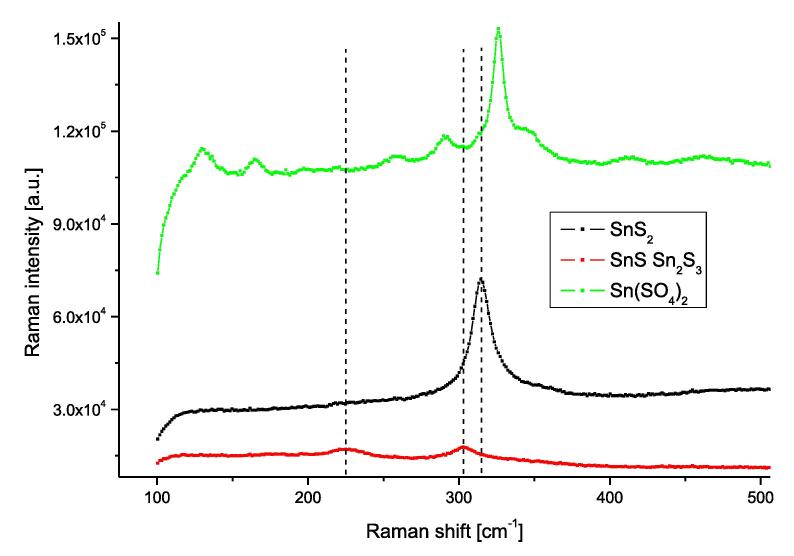
Typical Raman spectra of as-synthesized tin chalcogenide samples. Vertical dashed lines were placed for frequencies 225 cm^−1^, 303 cm^−1^ and 315 cm^−1^.

Raman spectroscopy was used to confirm the phase composition of the obtained chalcogenide samples. Raman studies have shown that the samples obtained are homogeneous in terms of chemical composition. Three different Raman spectra, related to three different crystal phases of the samples, were observed (Fig. 4). They correspond respectively to the polycrystalline structure of SnS_2_, SnS with the addition of the Sn_2_S_3_ phase, and Sn(SO_4_)_2_ (Fig. 4). Within a given type, the spectra differed only in the width of the peaks or their mutual intensity.

For most samples, the Raman spectrum corresponded to the SnS_2_ rhombohedral structure, with a characteristic peak of A_1g_ symmetry for about 315 cm^−1^ [39], [40]. The width of this peak is a measure of the life time of the corresponding phonon and indicates the size of the crystal grains and / or their structural quality. A smaller full width at half maximum indicates better structural quality of the sample and / or larger grain size for nanocrystalline structure. Table 3 shows the average peak width A_1g_ for each sample for which the Raman spectrum corresponded to the SnS_2_ structure.

**Table 3:** The full width at half maximum of the Raman A_1g_ peak at 315 cm^−1^ for different tested samples. The designation of samples is the same as in Table 1.

Sample	1	2	3	4	5	6	7	8	9
Width [cm^−1^]	17.6	15.4	16.8	18.5	14.7	20.5	14.2	23.9	18.9

For samples designated as 10, 12, 13 (according to Table 1) the Raman spectrum was in the shape represented by the red line in Fig. 4. The low intensity of this type of spectra is the result of high absorption by the sample of electromagnetic radiation in the studied spectral range. This spectrum has two characteristic, wide peaks located at about 225 cm^−1^ and 305 cm^−1^. The first peak correlates with the A_g_ mode for the SnS structure, while the second is characteristic of Sn_2_S_3_. The peak at 225 cm^−1^ comes from inter atomic vibration between metal (Sn) and chalcogen (S). The peak at 305 cm^−1^ is associated with the intralayer vibration of chalcogen–chalcogen ions [38]. In the case of nanostructures with SnS stoichiometry, the literature data shows the presence of both peaks, both for 225 cm^−1^ and for about 305 cm^−1^ [41]. It can therefore be concluded that the tested samples have a nano-grain structure with the SnS composition.

In the case of samples numbered 11, 14, 15 (according to Table 1) no peak characteristic of any tin sulfide phase was observed in the Raman spectra, the green line in Fig. 4. The Raman spectrum for these samples corresponds to the Sn(SO_4_)_2_ phase.

**Fig. 5:**
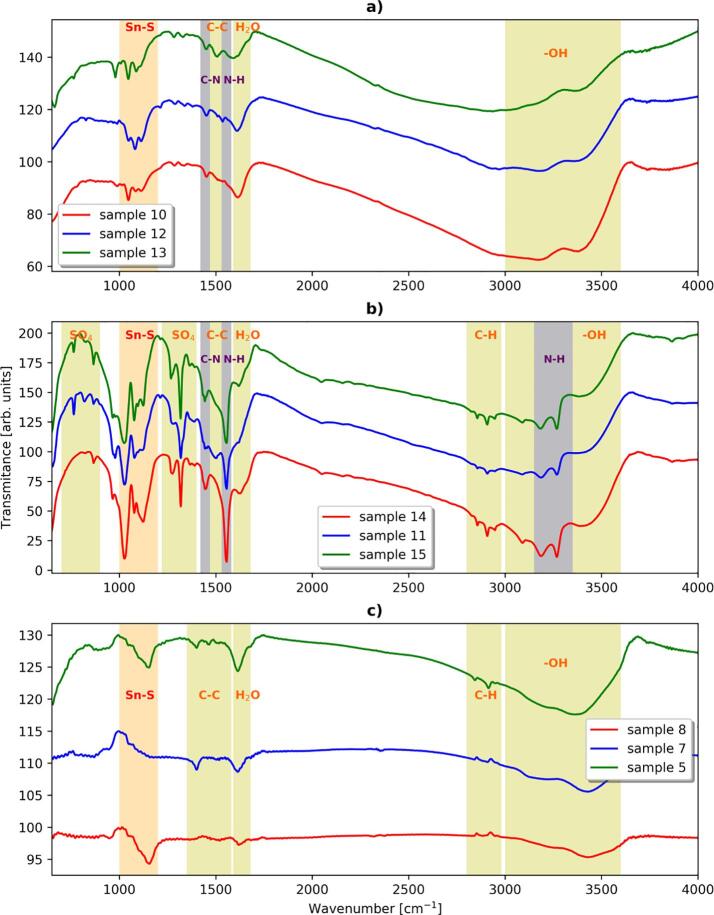
FTIR spectra of sonochemically prepared powders of: a) SnS, b) Sn(SO_4_)_2_, and c) SnS_2_. The samples are denoted as in Table 1.

Further structural characterization is performed by using the FTIR spectroscopy. FTIR spectra of prepared samples are presented in Fig. 5, together with typical ranges of wavenumbers corresponding to different chemical bonds. These ranges are taken from the literature [34], [42], [43], [44]. FTIR spectroscopy confirms the presence of Sn-S bonds in all prepared powders. It also turns out that each obtained powder contains some amount of water (from syntheses) and ethanol (from syntheses and purification). In the case of syntheses carried out in ethylenediamine, this solvent is also present in products (Fig. 5a) and 5b)). FTIR spectroscopy gives further justification of obtaining of Sn(SO_4_)_2_ in some cases, as revealed by occurrence of signals corresponding to SO_4_ groups (Fig. 5 b)).

From a chemical point of view it is intriguing that apart from the tin source used, every synthesis in ethanol gives SnS_2_, while sonochemistry in ethylenediamine is more diverse. Firstly, ethylenediamine protects Sn^2+^ from oxidation to Sn^4+^, partially at TAA:SnCl_2_ ratio 2.5 and strongly at TAA:SnCl_2_ ratio 3.5. Secondly, attempts to obtain powder of tin sulfide starting from SnCl_4_ in ethylenediamine failed. Thirdly, the sonochemistry in ethylenediamine soundly depends on TAA:tin source ratio: in the case of SnCl_4_ it is possible to drive the reaction to production of Sn(SO_4_)_2_ (white powder) or green perspicuous mixture; in the case of SnCl_2_ interplay between sonication time and TAA:tin source molar ratio determines which sulfide, SnS or SnS_2_, is obtained.

Under ultrasound irradiation thioacetamide decomposes to form, among other products, H_2_S which then reacts with tin cations forming corresponding tin sulfides:

Sn^2+^ + H_2_S → SnS + 2H^+^

Sn^4+^ + 2 H_2_S → SnS_2_ + 4H^+^

It requires detailed studies to determine the intrinsic mechanism of protection of Sn^2+^ oxidation in ethylenediamine. Hypothetically, this phenomenon may occur due to the complexation of Sn^2+^ cations by ethylenediamine or due to different mechanisms of thioacetamide degradation in ethanol and ethylenediamine. Moreover, the situation is further complicated because various byproducts can be formed during the whole process due to the presence of H^∙^ and OH^∙^ (and other) radicals in the reaction mixture, which are products of water sonolysis [26]:

H_2_O → H^∙^ + OH^∙^

**Fig. 6:**
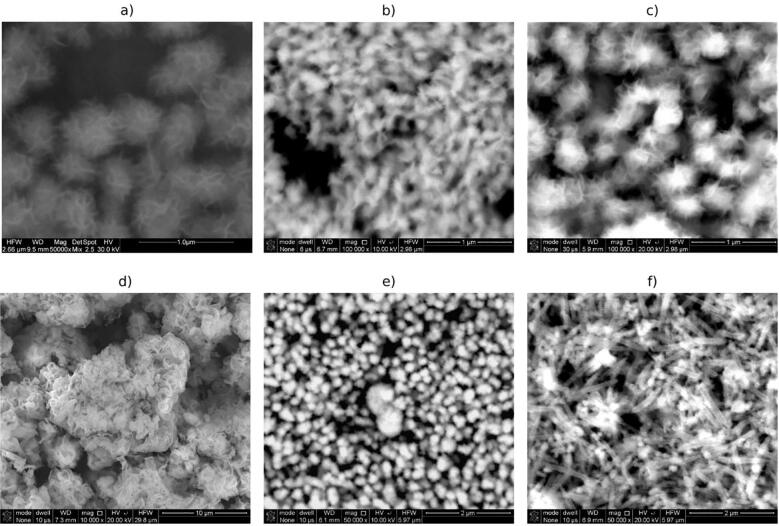
SEM images of powders obtained under different conditions (tin source, solvent, time, TAA:tin source ratio, product): a) SnCl_2_, ethanol, 160 min, 2.5, SnS_2_; b) SnCl_2_, ethanol, 100 min, 2.5, SnS_2_; c) SnCl_2_, ethanol, 230 min, 1, SnS_2_; d) SnCl_4_, ethanol, 100 min, 2.5, SnS_2_; e) SnCl_2_, ethanol, 100 min, 3.5, SnS_2_; f) SnCl_4_, ethanol, 100 min, 3.5, SnS_2_.

**Fig. 7:**
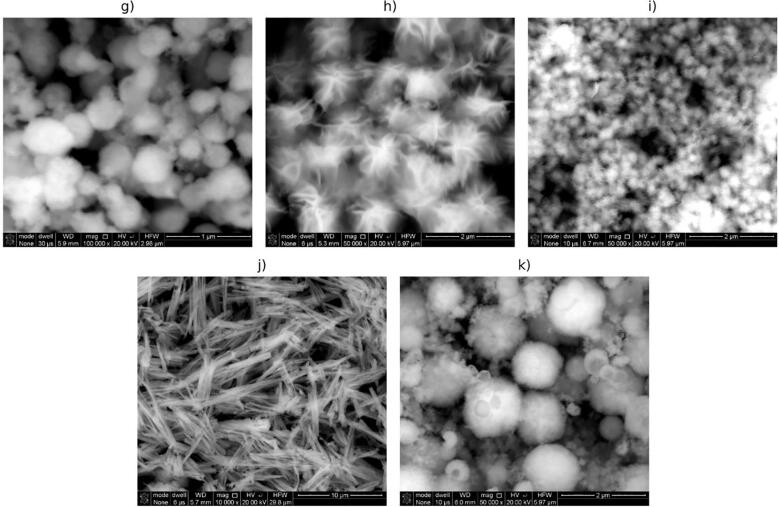
SEM images of powders obtained under different conditions (tin source, solvent, time, TAA:tin source ratio, product): g) SnCl_2_, ethylenediamine, 100 min, 3.5, SnS; h) SnCl_4_, ethanol, 160 min, 2.5, SnS_2_; i) SnCl_2_, ethanol, 160 min, 3.5, SnS_2_; j) SnCl_2_, ethylenediamine, 160 min, 2.5, SnS_2_; k) SnCl_2_, ethylenediamine, 160 min, 3.5, SnS.

Obtained tin(II) and tin(IV) sulfides are characterized by a variety of shapes and sizes, as revealed by scanning electron microscopy observations (Fig. 6, Fig. 7). Taking into account the type of detector used into SEM examinations the observed structures (Fig. 6, Fig. 7g-j) exhibits chemical homogeneity without no significant changes in chemical composition all of particular samples.

As can be seen in Fig. 6, SnS_2_ in ethanol can be obtained in the forms of flower-like submicron- and nanoparticles (images a), b), c), e) in Fig. 6), micro rods (image f) in Fig. 6) and in the form of random larger agglomerates without characteristic morphology (image d) in Fig. 6).

In Fig. 7 are presented more images showing flower-like morphology of SnS_2_ particles obtained in ethanol during ultrasound irradiation (images h) and i) in Fig. 7). Tin(IV) sulfide powder produced using ethylenediamine as a solvent consist of particles in needle shape (image j) in Fig. 7). SnS particles obtained in sonochemical syntheses in ethylenediamine showed globular morphology with a diameter up to c.a. 1 μm (images g) and k) in Fig. 7). However, the product of longer synthesis (160 min), starting from SnCl_2_ at TAA:SnCl_2_ ratio, is less uniform in size than the product of shorter synthesis (100 min) – see images k) and g) respectively in Fig. 7.

**Fig. 8:**
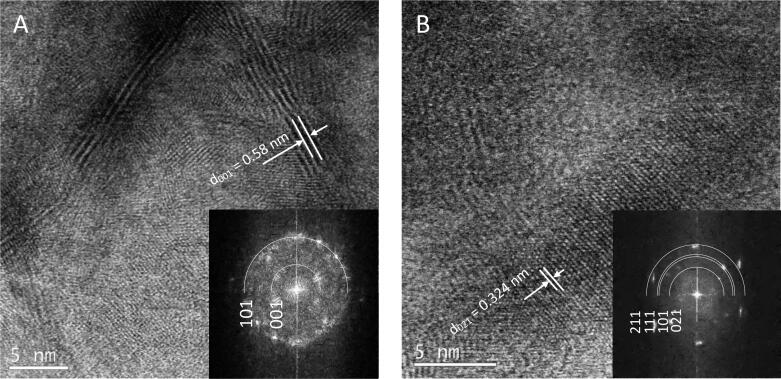
High resolution BF-STEM images of powders obtained under different conditions (tin source, solvent, time, TAA:tin source ratio, product): a) SnCl_2_, ethanol, 160 min, 3.5, SnS_2_; b) SnCl_2_, ethylenediamine, 100 min, 3.5, SnS.

The high resolution BF-STEM images were taken to confirm crystalline structure of the powders and phase analysis. Two powders were chosen for observations. The powders are mixture of very fine grains particles, which creates complex agglomerates therefore the observation has to be done carefully in specific places on the sample. The Fig. 8A shows a elongated shape particles of SnS_2_ phase with characteristic d_001_ = 0.58 nm lattice distance. The agglomerates consist also a mixture of more fine grains with size in the range 2–5 nm diameter. The Fig. 8B shows a different structure of the powder, identified as SnS. The bigger crystalline particle is surrounded by the amorphous phase.

**Fig. 9:**
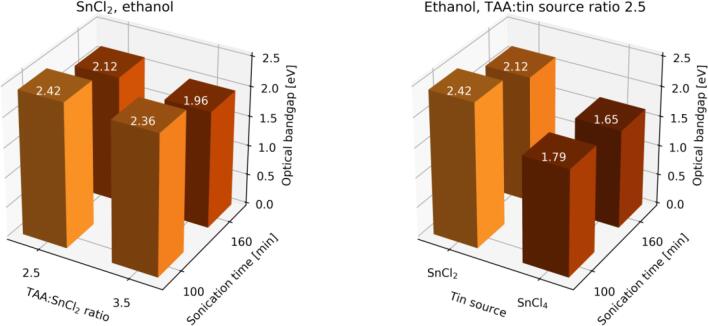
Comparison of optical bandgap values for products of syntheses: (left) conducted using SnCl_2_ as tin source and ethanol as solvent varying sonication time and TAA:SnCl_2_ ratio, and (right) conducted in ethanol and at TAA:tin source ratio 2.5 varying sonication time and tin source.

According to data presented in Table 1, the optical bandgap of sonochemically synthesized tin sulfides (both SnS and SnS_2_) decreases with the duration of synthesis. Moreover, the increase of TAA:SnCl_2_ ratio from 2.5 to 3.5 causes the decrease of the value of optical bandgap either using ethanol and ethylenediamine as the solvent. The same change in TAA:SnCl_4_ ratio causes the increase of optical bandgap value. The change of tin source from SnCl_2_ to SnCl_4_ also influences the value of optical bandgap causing its decrease. Fig. 9 shows comparisons of bandgap values for different synthesis conditions. The changes in bandgap values may be caused by many factors, including phase composition (some powders have relatively small amount of additional phase of other tin sulfide), formation of tin oxides layers on the surface of particles (especially in the case of SnS particles which are likely covered by layer of SnO), and the so-called quantum size effect responsible for widening of bandgap in nanomaterials.

## 4 Conclusions

This study investigates the sonochemical syntheses of SnS and SnS_2_ powders. The influence of different conditions of syntheses on properties of obtained products is examined. It was found that sonochemistry depends on used solvent – in ethanol mainly SnS_2_ is obtained, while in ethylenediamine one can obtain either SnS and SnS_2_, and even other compounds, depending on other parameters. Raman studies confirmed obtaining homogeneous tin (IV) sulphide samples in most cases. Samples with SnS stoichiometry additionally showed the presence of the Sn_2_S_3_ phase in Raman studies. The morphology and size of obtained powders also widely varies with synthesis conditions – from globular through flower-like to needle shape and from nano- to micropowder. It was also found that the longer sonication time increases the crystallinity of the product and decreases the value of optical bandgap. The sonochemical synthesis of tin sulfides allows obtaining powders with varied properties what may be technologically useful.

## CRediT authorship contribution statement

**Grzegorz Matyszczak:** Conceptualization, Formal analysis, Investigation, Methodology, Supervision, Visualization, Writing - original draft, Writing - review & editing. **Paweł Jóźwik:** Writing - review & editing, Visualization, Investigation, Formal analysis. **Emilia Polesiak:** Investigation, Formal analysis. **Małgorzata Sobieska:** Investigation, Formal analysis. **Krzysztof Krawczyk:** Supervision. **Cezariusz Jastrzębski:** Writing - review & editing, Visualization, Investigation, Formal analysis. **Tomasz Płociński:** Writing - review & editing, Visualization, Investigation, Formal analysis.

## Declaration of Competing Interest

The authors declare that they have no known competing financial interests or personal relationships that could have appeared to influence the work reported in this paper.
